# Supplementary material for: The causal role of circulating inflammatory markers in osteoporosis: a bidirectional Mendelian randomized study
Source: Front Immunol. 2024 Jul 18;15:1412298. doi: 10.3389/fimmu.2024.1412298 (PMC11291241; doi:10.3389/fimmu.2024.1412298)
Supplement: Supplementary file 1 [file DataSheet_1.zip › supplementary material/CIM-OSTEOPOROSIS /GCST90274824.csv_forest.pdf]

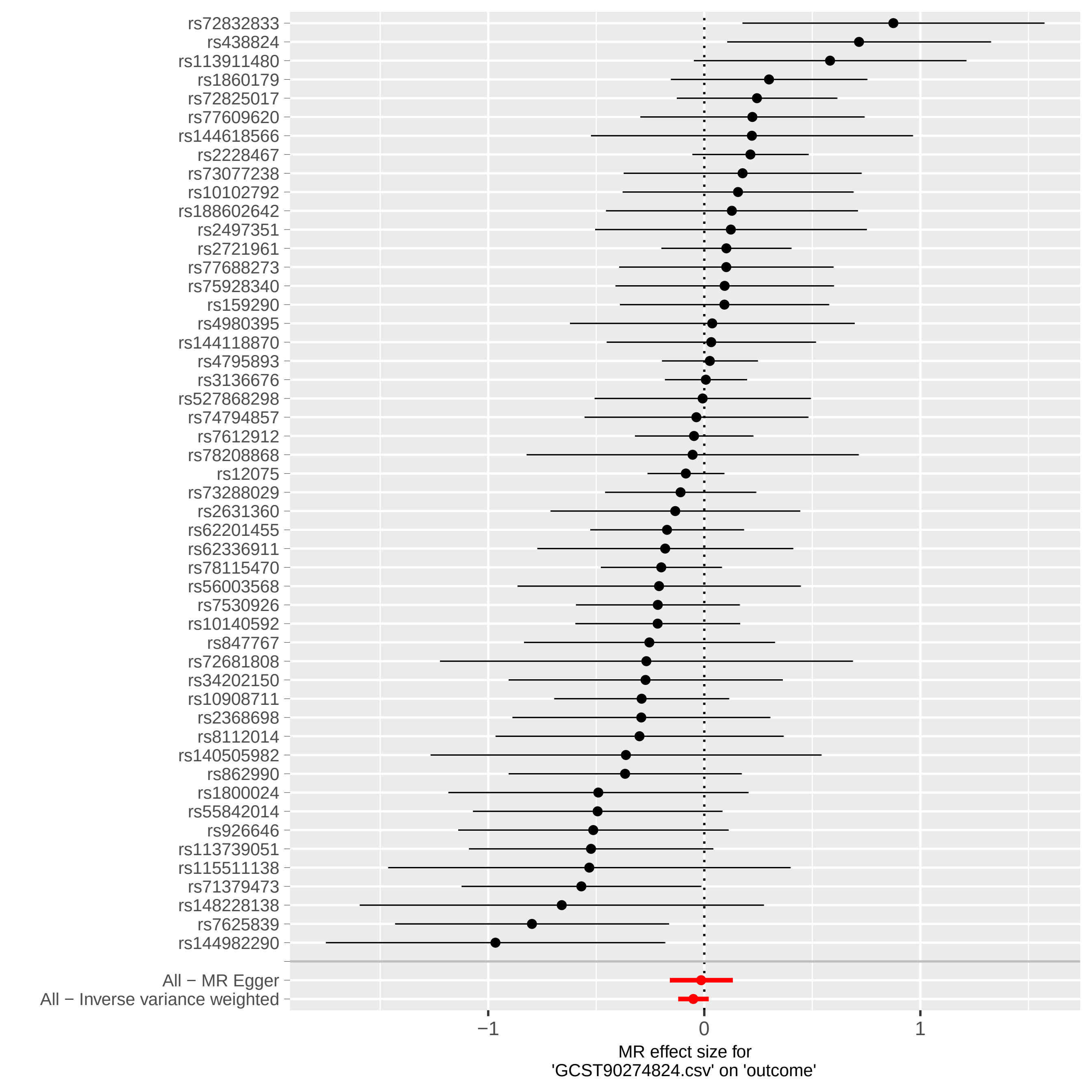

rs72832833
rs438824
rs113911480
rs1860179
rs72825017
rs77609620
rs144618566
rs2228467
rs73077238
rs10102792
rs188602642
rs2497351
rs2721961
rs77688273
rs75928340
rs159290
rs4980395
rs144118870
rs4795893
rs3136676
rs527868298
rs74794857
rs7612912
rs78208868
rs12075
rs73288029
rs2631360
rs62201455
rs62336911
rs78115470
rs56003568
rs7530926
rs10140592
rs847767
rs72681808
rs34202150
rs10908711
rs2368698
rs8112014
rs140505982
rs862990
rs1800024
rs55842014
rs926646
rs113739051
rs115511138
rs71379473
rs148228138
rs7625839
rs144982290
All − MR Egger
All − Inverse variance weighted
−1
0
1
MR effect size for
'GCST90274824.csv' on 'outcome'
